# Effectiveness of deep dry needling versus manual therapy in the treatment of myofascial temporomandibular disorders: a systematic review and network meta-analysis

**DOI:** 10.1186/s12998-023-00489-x

**Published:** 2023-11-03

**Authors:** Ángela Menéndez-Torre, Aitor Martín Pintado-Zugasti, Juan Nicolás Cuenca Zaldivar, Paula García-Bermejo, Diego Gómez-Costa, Miguel Molina-Álvarez, Alberto Arribas-Romano, Josué Fernández-Carnero

**Affiliations:** 1 Escuela Internacional de Doctorado, Department of Physical Therapy, Occupational Therapy, Rehabilitation and Physical Medicine, Universidad Rey Juan Carlos, 28933 Alcorcón, Spain; 2 Servicio de Fisioterapia, Centro Médico Gava, 28600 Navalcarnero, Madrid, Spain; 3 Departamento de Fisioterapia, Facultad de Medicina, Universidad San Pablo-CEU, CEU Universities, 28008 Madrid, Spain; 4 Rehabilitation Service, Guadarrama Hospital, Madrid, Spain; 5 Research Group in Nursing and Health Care, Puerta de Hierro Health Research Institute - Segovia de Arana (IDIPHISA), Madrid, Spain; 6 DINAMIA Clinic. Alfonso VI, 28806 Alcalá de Henares, Madrid, Spain; 7 Faculty of Sport Sciences, Universidad Europea de Madrid, Villaviciosa de Odón, 28670 Madrid, Spain; 8 Department of Nursing and Estomatology, Faculty of Health Sciences, Rey Juan Carlos University, Avenida de Atemas s/n, Alcorcón, 28922 Madrid, Spain; 9 Area of Pharmacology, Nutrition and Bromatology, Department of Basic Health Sciences, Universidad Rey Juan Carlos, Unidad Asociada I+D+i Instituto de Química Médica (IQM) CSIC-URJC, Madrid, Spain; 10 Department of Physical and Occupational Therapy, Rehabilitation and Physical Medicine, Universidad Rey Juan Carlos, 28922 Madrid, Spain; 11 La Paz Hospital Institute for Health Research, IdiPAZ, 28046 Madrid, Spain; 12 Grupo Multidisciplinar de Investigación y Tratamiento del Dolor, Grupo de Excelencia Investigadora, Universidad Rey Juan Carlos-Banco de Santander, 28922 Madrid, Spain; 13 Motion in Brains Research Group, Institute of Neuroscience and Sciences of the Movement (INCIMOV), Centro Superior de Estudios Universitarios La Salle, Universidad Autónoma de Madrid, 28023 Madrid, Spain

**Keywords:** Dry needling, Temporomandibular disorder, Musculoskeletal manipulations, Myofascial pain syndromes, Physical therapy modalities, Meta-analysis

## Abstract

**Background:**

Temporomandibular disorders (TMDs) are the most common cause of orofacial pain of non-dental origin, with approximately 42% of diagnoses corresponding to myofascial pain. Manual therapy and dry needling are commonly used interventions for the treatment of myofascial temporomandibular disorders. However, it is unclear whether one of them could be superior to the other.

**Objectives:**

The aim of the present systematic review and network meta-analysis was to compare the effectiveness of manual therapy and dry needling in patients with myofascial TMD.

**Methods:**

This is a systematic review and network meta-analysis. Randomized clinical trials were searched in the databases of Pubmed, PEDro, CINAHL, Web of Science, Scopus, Cochrane, Google Academic and EMBASE. The methodological quality of studies included in this review was judged using the Physiotherapy Evidence Database (PEDro) scale. A frequentist network meta-analysis was carried out, assuming random effects, to estimate the effects of interventions for temporomandibular joint pain measured on a 10-point visual analogue scale.

**Results:**

Out of 3190 records identified, 17 met the inclusion criteria for qualitative analysis and eight were included in the network meta-analysis. Indirect comparisons between dry needling and manual therapy showed no significant differences in their effects on pain reduction (Odds Ratio [95%CI]; − 0.263 [− 1.517, 0.992]). The ranking of treatments shows that manual therapy (SUCRA = 0.932) followed by deep dry needling (SUCRA = 0.775) present the highest values of estimation and can be considered the most likely to reduce pain.

**Conclusions:**

The results of the network meta-analysis should be considered with caution due to the low quality of the evidence available and the high variability of the study protocols in terms of the method of application of dry needling and manual therapy interventions.

PROSPERO under identifier: (CRD42020186470).

**Supplementary Information:**

The online version contains supplementary material available at 10.1186/s12998-023-00489-x.

## Introduction

Temporomandibular disorder (TMD) is the term generally used to describe the manifestation of pain and/or dysfunction of the temporomandibular joint and its associated structures [1]. These dysfunctions are primarily characterized by pain, joint sounds and limitation of jaw function [2]. TMD is an important problem that affects approximately 5% to 12% of the population. It can also affect activities of daily living, psychosocial functioning and quality of life [3].

TMD is classified as intra-articular if within the joint, with displacement of the articular disc involving the condyle–disc relationship, degenerative joint disease or subluxation; or extra-articular if the surrounding musculature is involved [4, 5]. The Diagnostic Criteria for Temporomandibular Disorders (DC/TMD) describe myalgia as pain of muscle origin that is affected by jaw movement, function, or parafunction, and replication of this pain occurs with provocation testing of the masticatory muscles. It is considered as one of the most common pain-related TMDs and is classified as local myalgia or myofascial pain in the case of pain spreading beyond the site of palpation [5].

Patients with myofascial TMD present myofascial trigger points (MTrPs) in the neck and masticatory muscles, which are thought to be implicated in the pathophysiology and manifestations of myofascial TMD [6]. MTrPs are hypersensitive locations in a taut band of skeletal muscle or muscle fascia that can cause numerous sensory, motor, neurological and autonomic symptoms [7]. Previous observational research reported that myofascial pain was present in 42% of patients with TMD, being the most frequent diagnosis in this population [8].

Various types of therapeutic interventions have been investigated to treat MTrPs, including invasive (e.g. dry needling or acupuncture) and non-invasive treatments (e.g. manual therapy, electrotherapy, exercise, occlusal splints). In previous systematic reviews, both types of treatments have shown its effectiveness at reducing pain symptoms in patients with TMD [9, 10]. Manual therapy for TMD includes soft tissue and joint mobilization, active or passive stretching, exercises, manual compression and massage [11, 12]. Despite the numerous existing studies on manual therapy for TMD, a recent systematic review reported the need for further research due to the variability, methodological limitations, inconclusive data and poor homogeneity of existing investigations [13].

Dry needling consists of the insertion of a solid sterile fine needle through the skin to stimulate the MTrP [14]. Dry needling of active MTrPs in the masseter and temporalis muscles in patients with myofascial TMD has been found to produce immediate improvements in and relief of pain, tenderness and function [15]. The low quality of evidence and the high risk of bias of some studies included in a recent systematic review suggested that larger trials with a lower risk of bias are needed to evaluate the effects of dry needling on myofascial TMD [10].

Dry needling is a common intervention in clinical practice nowadays, but its effectiveness compared to other conservative treatment modalities, such as manual therapy, remains unclear. Therefore, this comparison has been the focus of previous research in patients with neck pain in order to provide further insight into whether any of these therapies could be considered superior to the other [16–18]. From a clinical perspective, sometimes the rationale for the selection of any of these two therapies is not clear and they are used indistinctively. A previous network meta-analysis (NMA) assessed the hierarchy of twelve different treatment modalities for patients with myofascial TMD and showed manual therapy as the most effective treatment [19]. However, the authors recommended cautious interpretation of the results due to study limitations and the scarcity of strong evidence. Several randomized controlled trials investigating the effectiveness of manual therapy or dry needling for myofascial TMD have been published after the search performed in the above-mentioned NMA [20–23]. Therefore, there is a need to update the evidence regarding the effects of these techniques and their comparison.

Dry needling and manual therapy are two of the treatments that have demonstrated the effect in improving pain and function in patients with myofascial TMD [19]. In a recent NMA, they concluded that although there is not enough support for dry needling, this technique does significantly decrease pain intensity compared to placebo [19].

### Objective

The aim of the present systematic review and NMA was to compare the effectiveness of manual therapy and dry needling in patients with myofascial TMD, in terms of pain at rest, pressure pain threshold (PPT), pain on chewing, disability and mouth opening range.

## Materials and methods

### Protocol and registration

This NMA was designed according to the Preferred Reporting Items for Systematic Reviews and Meta-Analyses (PRISMA) statement and PRISMA extension for NMA (PRISMA-NMA) [24, 25]. It was registered on PROSPERO (CRD42020186470).

Initially, the review was registered as a pairwise meta-analysis in PROSPERO with date 28/05/2020. As no randomized controlled trials (RCTs) comparing manual therapy and dry needling were found in this first phase, it was decided to perform a NMA to indirectly compare these two therapies. The appropriate changes were made in the PROSPERO registry.

### Search strategy

All RCTs were selected through extensive research in PUBMED, PEDro, Web of Science, CINAHL, Cochrane, Scopus, Google Academic and EMBASE during May and June, 2020. The search strategy was adapted to the different default options in each database based on the combination of keywords and Booleans included in Additional file 1: Appendix S1.

### Study eligibility criteria

For inclusion in this systematic review, the articles had to fulfil the following criteria: (1) any type of RCT, (2) sample of human adults (age ≥ 18 years), (3) including dry needling and/or manual therapy in any of the study groups, (4) sample diagnosed with myofascial TMD according to the Diagnostic Criteria for Temporomandibular Disorders (DRC/TMD) [5] or according to the diagnosis of an active MTrP [7] and (5) measuring at least one of the following outcome measures: pain intensity, PPT, pain on chewing, disability or mouth opening range.

The following exclusion criteria were applied: (1) studies that included subjects under 18 years old, (2) subjects diagnosed with arthrogenous TMD, (3) studies in which co-interventions did not include any group of the above interventions, (4) crossover studies, single-arm designs, reviews, letters, case reports, case series, and (4) full paper not available.

### Study selection and data extraction

After database search, the results were imported to a reference manager software (Mendeley desktop v1.17.4, Elsevier, New York, New York) to remove duplicates automatically. Two independent researchers (A.M.T and P.G.B) used the previously described eligibility criteria to screen the records by title and abstract. Then, full-text articles were assessed for eligibility.

Data were extracted by the same two independent reviewers. Standardized data extraction was performed in order to select the following data: study characteristics (author and year), sample characteristics (size, sex and age), diagnosis, intervention, outcome measures, and timing of follow-up assessment and results (means and SDs for outcome measures at baseline and follow-up). If the values of mean and SD were not completely reported in the original article, the authors were contacted via email to request the data. Articles with a lack of data not receiving an answer from the authors were excluded from the meta-analysis. When the articles reported the results as median and quartile values, the mean and standard deviation were estimated according to previously described methods [26]. In studies in which the confidence intervals or standard error (SE) were reported instead of the standard deviation, this was calculated using the appropriate formulas [27].

In case of disagreements about the eligibility of a study or data extraction, the authors consulted a third author (A.M.P.Z) to reach a common decision through consensus.

The primary outcome measures considered for data extraction were pain intensity, PPT, pain on chewing, disability and mouth opening range. In order to be included in the NMA, the outcome measure had to have been assessed by at least three articles investigating the same intervention over a similar timescale (immediate, short term, medium term or long term).

### Quality assessment

The methodological quality of the studies was assessed according to the Physiotherapy Evidence Database (PEDro) scale by the two independent reviewers. The PEDro scale is a valid measure of the methodological quality of clinical trials [28]. It is based on the Delphi list developed by Verhagen and collaborators in the Department of Epidemiology, University of Maastricht [29].

The PEDro scale consists of eight items related to the methodological quality (random assignment, hidden assignment, comparability at the beginning, blinded participants, blinded therapists, blinded evaluators, adequate follow-up and intention-to-treat type analysis), two items related to statistical information (comparisons between groups and point estimates and variability) and one additional item (election criteria), which is not used for the calculation of the total score. The total score ranges from 0 to 10 points [30]. Studies are considered to be of acceptable quality when they meet six or more criteria [31].

### Statistical analyses

For the statistical analysis, the R Ver. 4.0.5 program (R Foundation for Statistical Computing, Institute for Statistics and Mathematics, Welthandelsplatz 1, 1020 Vienna, Austria) was used with the netmeta [32] and dmetar [33] packages. Where the median and interquartile range were shown, these were transformed into mean and standard deviation using the appropriate formulas [26, 34].

A frequentist NMA with Dersimonian-Laird estimator design was carried out assuming a random effects model for temporomandibular joint pain measured on a 10-point visual analogue scale (VAS) at follow-up using the mean difference (MD) as effect size measure.

A network diagram was created in which each node represents an intervention, and the effect of pairwise comparisons of two interventions is shown as lines interconnecting the nodes, where the thickness of the lines represents the weight of pairwise comparisons. The number of studies contributing to each pairwise comparison is shown on each line.

The assumption of transitivity was evaluated assuming that all the interventions analyzed present the same results regardless of the study to which they belong. To do this, it was verified that the confounding variables age and male/female ratio presented a similar distribution throughout the comparisons. For this, the graph of the structure of the network was created, weighting the size of the nodes by said covariates, visually evaluating in which comparisons the covariates were not balanced [35].

The presence of inconsistency was assessed using node-splitting and by analysing the level of significance (set at p < 0.05) of the Z statistic to detect disagreement between direct and indirect comparisons of each intervention. The contribution of individual studies to the network and its methodological quality was also analysed through the contribution matrix.

Heterogeneity was assessed by estimating the overall and decomposed within and between studies Cochrane´s Q test, as well as with the estimator I^2^ as a measure of the proportion of observed heterogeneity that is due to true heterogeneity between studies, rather than random error, which was defined as: 0–30%: unimportant heterogeneity; 30–50%: moderate heterogeneity; 50–75%: large heterogeneity; 75–100%: important heterogeneity.

Ranking of mixed (direct and indirect) effect sizes of the treatments was analysed using SUCRA (Surface Under the Cumulative Ranking Curve) accompanied by visual inspection of the rankogram and the league table for pairwise comparisons. These indicators show the likelihood that an intervention is more effective than the other interventions in the network.

Publication bias was analysed using a comparison adjusted funnel plot, and with the Egger, Begg–Mazumbar and Thompson–Sharp test.

## Results

### Study selection

The results of the database search showed that there were no previously published eligible RCTs directly comparing dry needling and manual therapy in independent groups. Instead, articles compared dry needling or manual therapy with placebo or with other therapies including botulinum toxin and types of cognitive therapy, such as information, education and self-care. Based on this research situation, the NMA included five comparative arms (deep dry needling, manual therapy, botulinum toxin, placebo and cognitive therapy) in order to indirectly compare the effects of dry needling and manual therapy, which was the primary objective of the present study.

A total of 17 RCTs evaluating the effectiveness of manual therapy or dry needling in the treatment of TMDs of myofascial origin were included in the systematic review, while eight of them were included in the NMA.

**Fig. 1:**
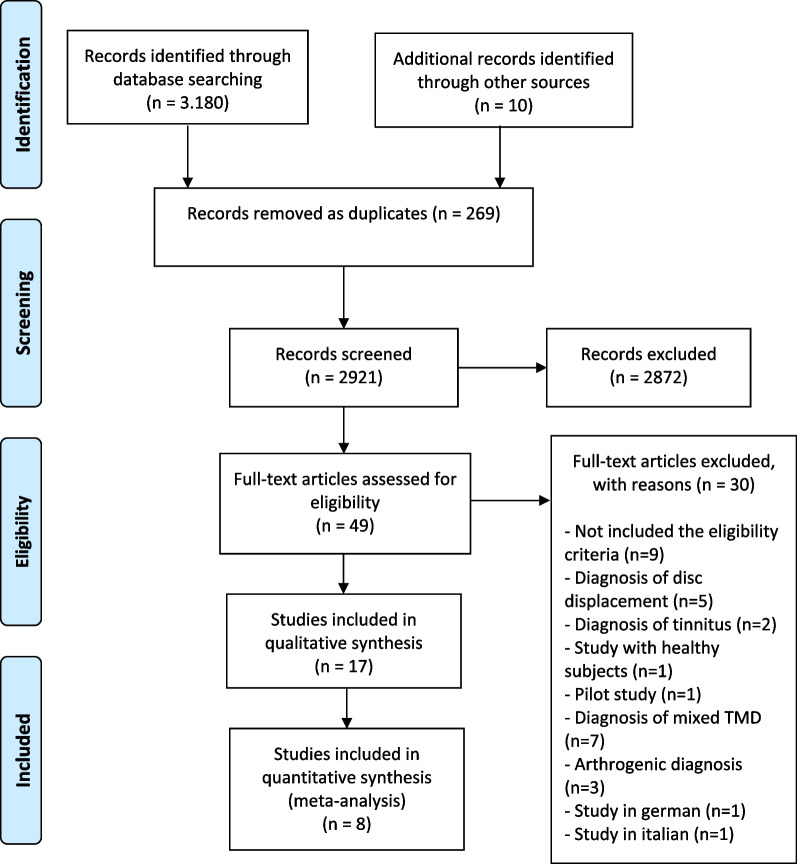
Flow of studies through the review

Although all the included studies were RCTs, there were variations in the methods used in terms of the number of sessions of each therapy, the follow-up time points or the outcome measures. The only outcome measure that was consistently evaluated in at least three studies of dry needling and three studies of manual therapy over the same timescale (short term: between 1 and 3 months) was pain intensity. Therefore, a total of eight RCTs were included in the NMA; five evaluated the effects of manual therapy [21, 22, 36–38] and three the effects of dry needling [20, 23, 39] on pain intensity between 1 and 3 months after the end of the treatment. The flow of studies through the selection process of the review is presented in Fig. 1.

### Study quality

According to the PEDro scale, with a theoretical maximum of 10 points and the 6/10 is the threshold for a high-quality study, 13 studies exceeded the limit of 6 and four showed a low quality level (< 6). The total PEDro score ranged from 4 to 9, with a mean of 6.71 (Additional file 2: Appendix S2).

### Study characteristics

Study characteristics and the results of individual studies are presented in Additional file 3: Appendix S3, including: (a) author and publication year; (b) sample; (c) treatment groups; (d) therapy; (e) number of treatment sessions; (f) follow-up time; (g) outcome measures and (h) main results.

Among the 17 studies included in the systematic review there was a total of 867 patients over 18 years old, of whom the majority were women. Of these participants, 258 received dry needling or were in the control groups of the studies in which needling was evaluated. The remaining 609 individuals belonged to the treatment or control groups of the manual therapy studies.

In the seven selected dry needling studies, the duration of the intervention ranged from one to three sessions. The dry needling techniques used varied in the selected studies: two performed dry needling in the lateral pterygoid muscle, two in the masseter muscle, two did not specify the exact muscle in which the technique was performed and one combined dry needling in the masseter and temporalis muscles.

In the 10 articles on manual therapy, there was substantial variability regarding the treatment modality and dosage. Moreover, seven performed manual therapy in isolation from the experimental group, while three combined manual therapy with other techniques.

Eleven studies had a control group, of which three received treatment, two received no intervention and six performed placebo techniques. One of the selected studies combined dry needling with another technique (simulated anaesthesia). The remaining six articles on dry needling performed this technique alone.

The most commonly measured variables in the selected studies were age, sex, pain at rest, pressure pain threshold, pain on mastication, functionality and mouth opening range.

## Network meta-analysis

### Presentation of network geometry

**Fig. 2:**
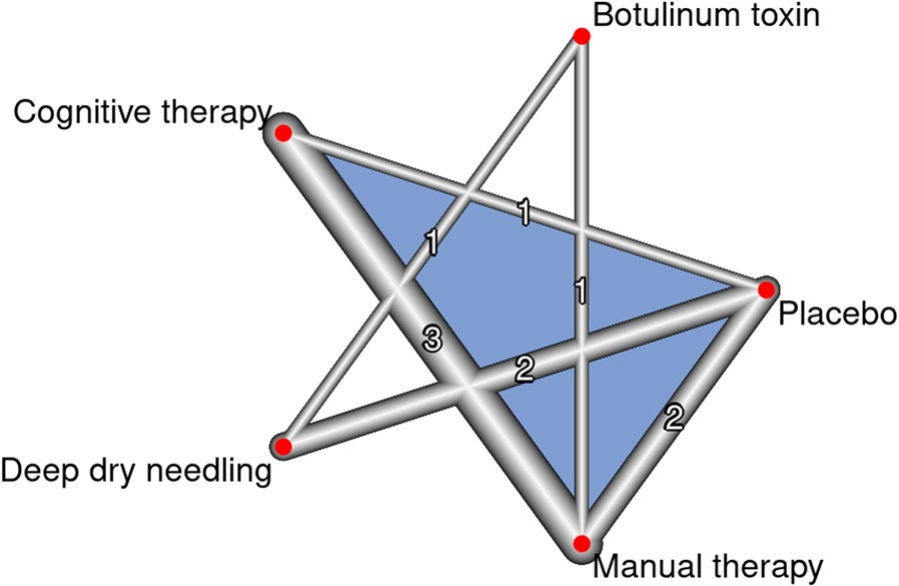
Network geometry for pain intensity in the short term

A total of eight studies were included in the NMA, including six designs with five interventions (deep dry needling, manual therapy, cognitive therapy, botulinum toxin and placebo) and 10 pairwise comparisons, including 556 patients (median ± IR, 47 ± 25 patients/study). The network graph shows that the largest number of studies compared manual therapy vs. cognitive therapy and placebo, as well as dry needling versus placebo (Fig. 2).

### Exploration for inconsistency/heterogeneity

The significant Cochrane’s Q test (X^2^(5) = 14.421, p = 0.013) and the I^2^ value of 65.327%, 95%CI ( 16.757%, 85.558%) indicated significant and large heterogeneity. On the other hand, intra-study heterogeneity (X^2^(2) = 7.953, p = 0.019) is significant while between-study inconsistency (X^2^(3) = 6.468, p = 0.091) is no significant; however, when consistency was evaluated under the assumption of a full random-effects model of design-by-treatment interaction, the between-study inconsistency decreases and remains non-significant (X^2^(3) = 3.213, p = 0.36).

The node-splitting method showed no significant differences in Z-scores (p > 0.05) between the direct–indirect estimates of the network, which seems to indicate sufficient consistency in the comparisons made (Additional file 4: Appendix S4).

The graph of direct and indirect comparisons shows a high percentage of indirect comparisons in the total estimate of each comparison. The comparisons cognitive therapy vs. manual therapy, dry needling vs. placebo, manual therapy vs. placebo and botulinum toxin vs. dry needling present an average length of connections greater than 2, indicating that they may be the cause of worse compliance with the assumptions of the model (Additional file 5: Appendix S5).

Regarding transitivity, the network graphs with the size of the interventions weighted by the covariates show how the age distribution is similar across the comparisons, while in the case of the male/female ratio, the manual therapy vs. botulinum toxin and vs. cognitive therapy and placebo vs. cognitive therapy and vs. dry needling cannot be assured that the effects in these comparisons are not influenced by this confounding variable (Additional file 6: Appendix S6).

### Synthesis of results

**Fig. 3:**
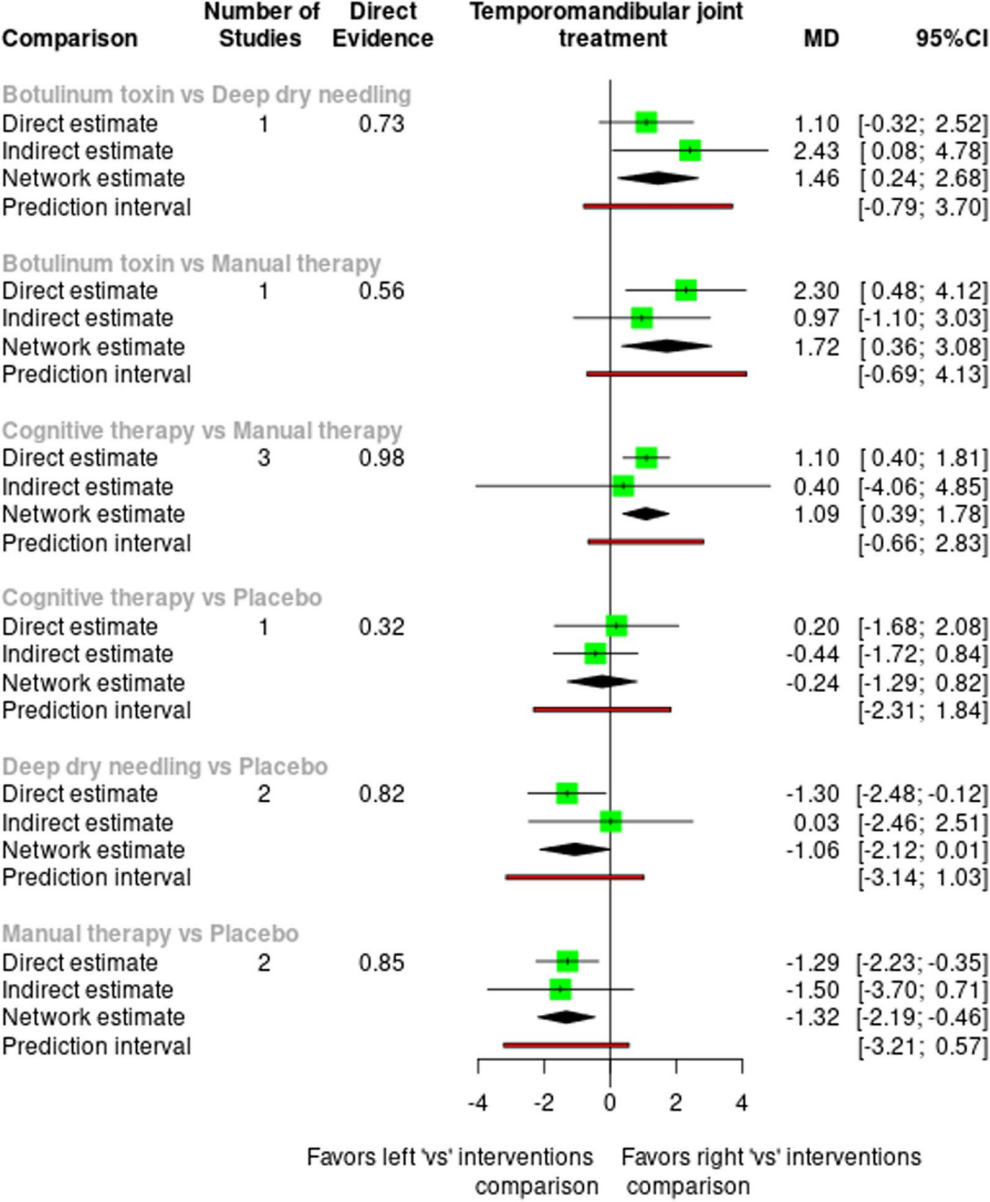
Split forest plot for pain intensity at the short term. SMD, standardized mean difference; CI, confidence interval

The split forest plot shows both direct and network significant differences between botulinum toxin vs. manual therapy, cognitive therapy vs. manual therapy and manual therapy vs. placebo in favor of manual therapy in all cases, and in the comparison in network botulinum toxin versus dry needling in favor of dry needling, and with a significant mean difference (Fig. 3).

**Fig. 4:**
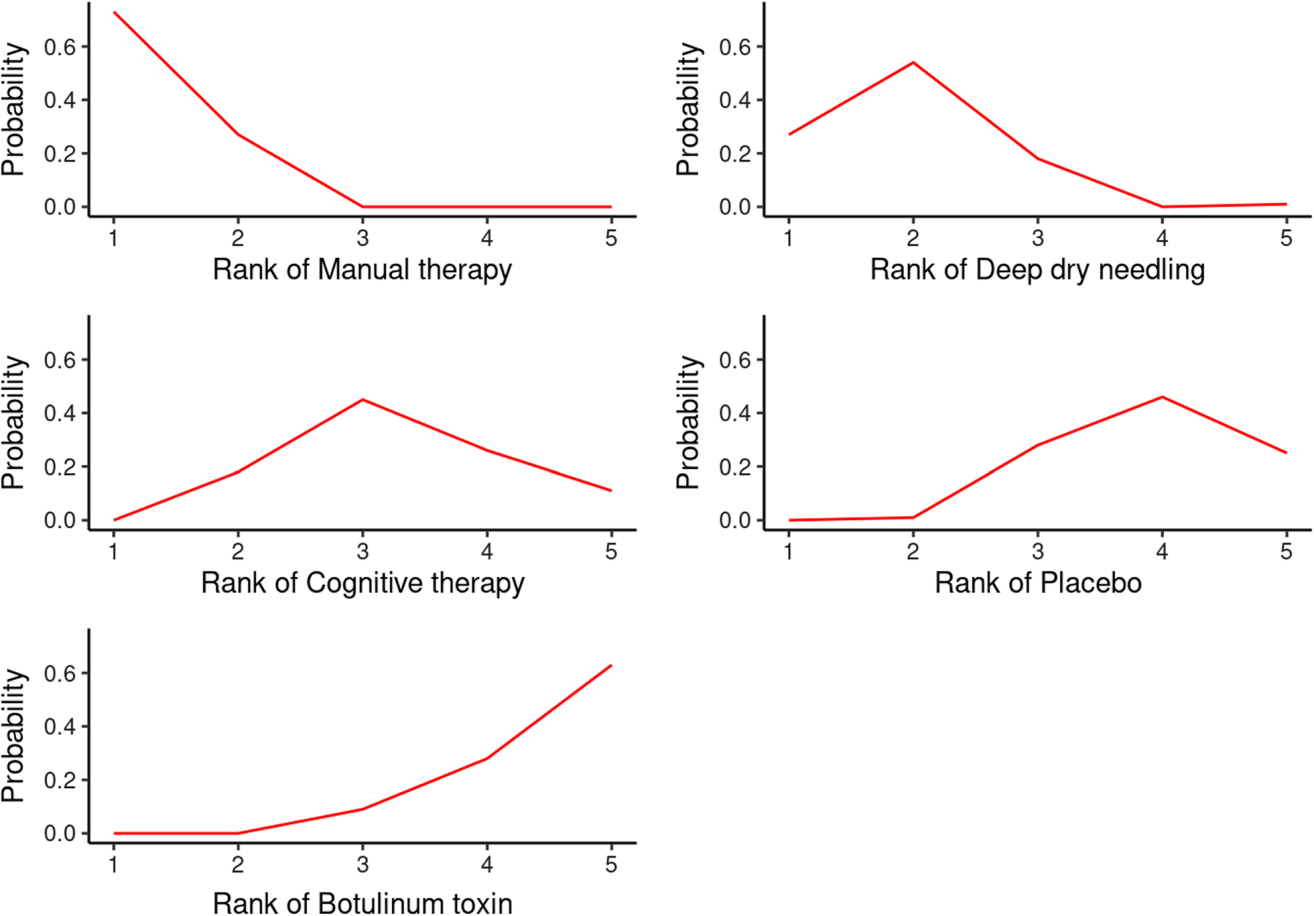
Rankogram contribution plot

The ranking of treatments shows how Manual therapy (SUCRA = 0.902) followed by Deep dry needling (SUCRA = 0.807) are, those that present the highest values of SUCRA estimation and can be considered the most likely to reduce pain, while Cognitive therapy (SUCRA = 0.398), Placebo (SUCRA = 0.29) and Botulinum toxin (SUCRA = 0.103) are the less likely to reduce pain. The rankogram once again shows manual therapy as the intervention with the highest probability of success in reducing pain, followed by dry needling (Fig. 4).

The contribution table shows that the studies with the greatest influence on the mixed comparisons are the studies by Gonzalez-Perez et al. [39] and Lopez-Martos et al. [20] that compared dry needling versus placebo (0.841%), and the studies by Corum [22], De Laat [36] and Kalamir [38] that compare cognitive therapy vs. manual therapy (0.897%). However, no direct comparison influenced more than 1% in the total of mixed comparisons, so it is unlikely that the methodological quality of the individual articles did not bias the consistency of the analysis (Additional file 7: Appendix S7).

The league table shows that the comparisons with a significant effect on pain reduction are manual therapy vs. cognitive therapy − 1.085 (− 1.782, − 0.389) vs. placebo − 1.322 (− 2.188, − 0.455) and vs. botulinum toxin − 1.720 (− 3.083, − 0.357), favouring manual therapy on the one hand, and dry needling vs. botulinum toxin − 1.457 (− 2.676, − 0.239), favouring dry needling on the other hand (Table 1).

**Table 1:** League table reporting the comparative effects for all interventions for the pain intensity network

Manual therapy				
− 0.263 (− 1.517, 0.992)	Deep dry needling			
− 1.085 (− 1.782, − 0.389)	− 0.822 (− 2.224, 0.579)	Cognitive therapy		
− 1.322 (− 2.188, − 0.455)	− 1.059 (− 2.124, 0.006)	− 0.237 (− 1.292, 0.819)	Placebo	
− 1.720 (− 3.083, − 0.357)	− 1.457 (− 2.676, − 0.239)	− 0.635 (− 2.146, 0.877)	− 0.398 (− 1.767, 0.970)	Botulinum toxin

Ordered by SUCRA

**Fig. 5:**
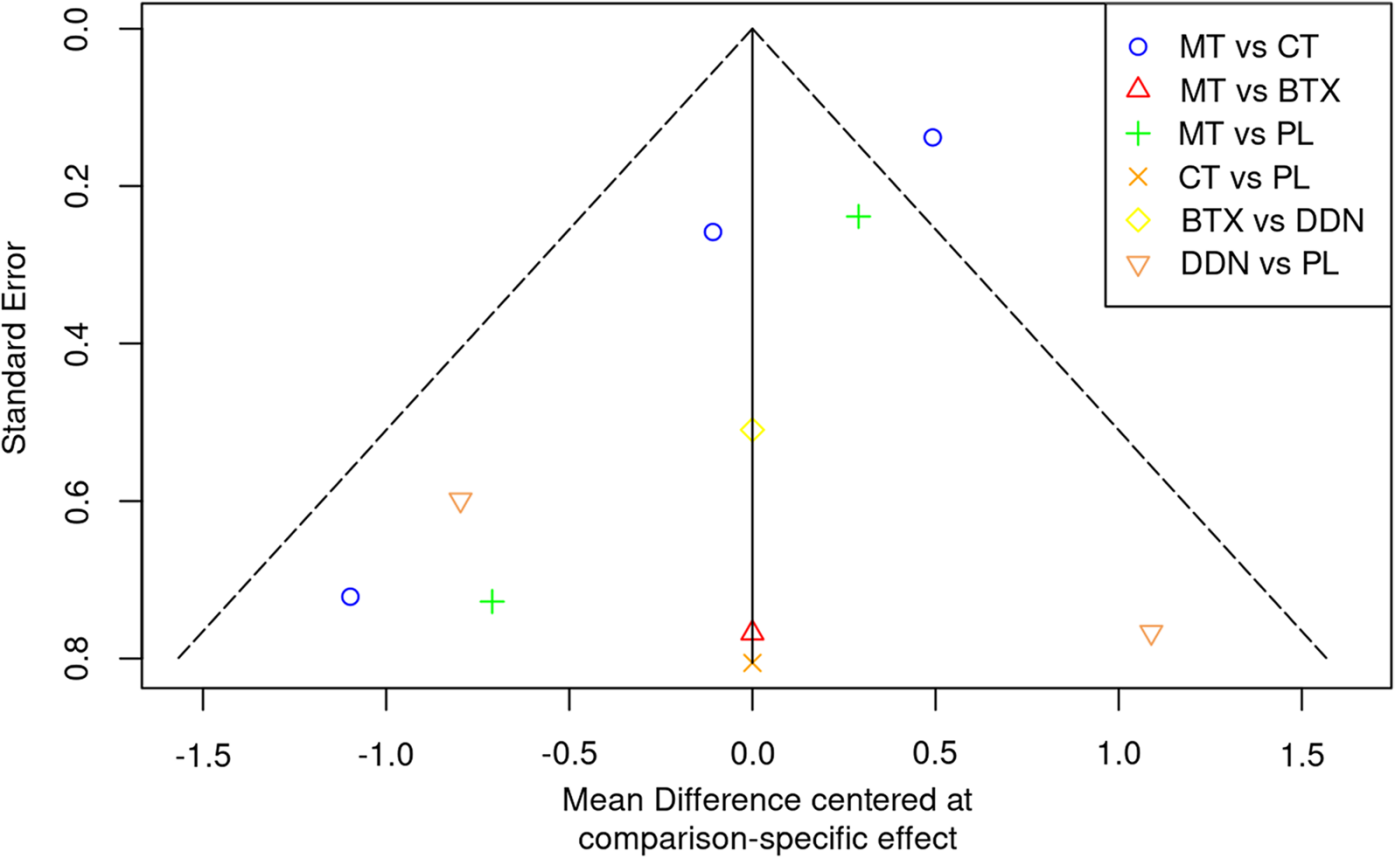
Publication bias funnel plot. PL, Placebo; CT, Cognitive therapy; MT, Manual Therapy; DDN, Deep dry needling; BTX, Botulinum toxin

Despite Egger’s tests (t(8) =  − 2.269, p = 0.053), Begg–Mazumbar (Z = − 0.268, p = 0.788) and Thompson–Sharp (t(8) =  − 2.008, p = 0.079) not significant, the funnel plot presents studies outside the significance bands and distributed asymmetrically around the null axis, which indicates the presence of bias of publication (Fig. 5).

## Discussion

This systematic review and meta-analysis of eight trials (n = 556 subjects) investigated the short-term effectiveness of dry needling compared to manual therapy and other pain management interventions in patients with temporomandibular pain of myofascial origin. Initially, the aim was to compare the effects of manual therapy and dry needling for multiple outcomes and at different follow-up time points. However, the characteristics of the published studies only allowed investigating the pain outcome in the short term (1–3 months) and not an immediate, medium-term or long-term follow-up. All patients, except those included in the study by Kütük et al. [23], were clinically examined and diagnosed with TMD according with the Research Diagnostic Criteria for Temporomandibular Disorders (RDC/TMD) guidelines [5]. Kütük et al. [23] examined patients according to the criteria for the diagnosis of MTrP [7].

We found evidence that manual therapy has a greater effect on pain reduction than placebo, botulinum toxin and cognitive therapy, and that dry needling has a significant effect on pain reduction than botulinum toxin. Indirect comparisons between dry needling and manual therapy showed no significant differences in their effects on pain reduction. However, the results of the NMA showed manual therapy as the intervention with the highest probability of success in reducing pain, followed by dry needling. These results from the NMA should be viewed with caution given the small number of existing publications, the heterogeneity between articles and methodological limitations. Moreover, the comparisons between manual therapy vs. botulinum toxin and vs. cognitive therapy should be interpreted with caution due the potential influence of the male/female ratio as a confounding variable.

In line with our results, a previous recent meta-analysis investigating the efficacy of manual therapy for TMD found short- and medium-term improvements in pain, although the effects appeared to decrease over time [40]. The authors also highlighted the high variability regarding the modality of manual therapy applied in each study, including the muscles that were the target of the treatment, and whether the approach was intraoral or focused on joint manipulation therapy.

A previous NMA investigating the hierarchy of different treatments for myofascial TMD also showed manual therapy as the most effective treatment for pain reduction in the overall follow-up [19]. Dry needling ranked sixth out of ten different interventions. These results showed that manual therapy was the most effective treatment for myofascial TMD, which is in line with the results of the present NMA, despite the methodological differences between the two meta-analyses. In contrast, the treatment ranking in the previous meta-analysis showed that dry needling ranked lower than botulinum toxin. Differences between the two reviews include that our database search was performed 2 years later than the first, so various RCTs from this period were only included in our meta-analysis. Moreover, there were also differences in the selection criteria used to include RCTs, such as the fact that the previous meta-analysis only included RCTs with a placebo or control group comparator or the fact that our meta-analysis used different follow-up time points on pain intensity.

Vier et al. [10] investigated the effects of dry needling versus placebo or other interventions (not including manual therapy) and suggested that dry needling is superior to placebo for PPT and superior to other interventions for pain intensity in the short term. However, the low quality of evidence and risk of bias limited the possibility of drawing strong conclusions about the effectiveness of dry needling. Another recent NMA comparing different needling therapies in the management of pain of the masticatory muscles concluded that the effectiveness of needling therapy did not depend on the needling modality (wet or dry) or the injected substance [41]. However, the low quality of evidence limited the possibility of providing enough support for any of the needling therapies.

Most studies included in this meta-analysis showed good methodological quality, but some of them showed a low level of methodological quality according to the PEDro scale (< 6). There is a need for further research through randomized controlled trials with larger samples investigating the effects of dry needling or manual therapy for the treatment of TMDs.

At present, there is a lack of RCTs comparing dry needling and manual therapy for the treatment of myofascial TMD. However, this comparison has been investigated through clinical trials in other pathologies and body regions [17, 42].

A recent study by Otadi et al. [43] compared the immediate and short-term effects of the combination of dry needling and education versus manual therapy (ischemic compression) combined with education to treat myofascial trigger points in subjects with neck pain. Both groups showed similar effects on immediate and short-term follow-up for relieving pain, but manual therapy resulted in faster positive results, suggesting higher effectiveness of the manual therapy group at reducing pain.

In a recent systematic review and meta-analysis, Lew et al. [16] compared dry needling and manual therapy to reduce pain and improve function in patients with neck pain, showing that neither therapy is superior to the other, since both improved pain and function in the short and medium term. The authors suggested that future research should evaluate the effects of dry needling and trigger point manual therapy (TPMT) against sham interventions on improving pain and function in different body regions, as well as further clarification of optimal dosages of dry needling and manual therapy.

In the present systematic review and NMA, it is also evident that there is substantial heterogeneity regarding the modality and dosage of both dry needling and manual therapy. Among the RCTs of dry needling included in the NMA, two of them applied deep dry needling in the lateral pterygoid for three sessions (one session/week), while the study of Kütük et al. [23] only applied one session without specifying the muscle receiving the intervention. Regarding the studies of manual therapy, none of them applied the same modality of intervention. For instance, Kalamir et al. [38] applied myofascial intraoral therapy, while Reynolds et al. [21] combined different therapies of thrust, inhibition of suboccipital muscles, exercise and education.

### Limitations

As in any systematic review, there is the possibility of selection bias; however, a comprehensive search strategy was used and included both database searching and manual searching. In addition, two independent researchers collaborated in the selection of articles, which limited the biases that a single person could have individually. On the other hand, the gray literature was not reviewed.

The small number of articles included in the review, as well as their heterogeneity in terms of follow-up time points, the differences in the number of treatment sessions, muscles treated or variability of manual therapy and dry needling techniques used, limit the relevance of the results of the NMA. Moreover, the clinical relevance that the significant differences obtained between therapies or the treatment ranking could have for patients regarding their pain intensity perception remains unclear.

At present, no RCTs have compared dry needling and manual therapy in the treatment of myofascial TMD, which limits the possibility of concluding which of the two techniques is more effective based on indirect comparisons.

Finally, it should be considered that the present review only focused on investigating the effects of individual therapies in isolation, since this may provide valuable information for clinicians in the reasoning process used to choose the most appropriate treatment approach. However, previous research suggested that multimodal approaches are probably the most effective in patients with TMDs or that the complementation of manual therapies with therapeutic exercise seems to be associated with larger treatment effects.

## Conclusion

Indirect comparisons between dry needling and manual therapy showed no significant differences in their effects on pain reduction in patients with myofascial TMD. However, manual therapy (SUCRA = 0.902) was the intervention with the highest probability of success in reducing pain in the short term, followed by dry needling (SUCRA = 0.807). Moreover, manual therapy had a significant effect on pain reduction than placebo (− 1.322), botulinum toxin (− 1.720) and cognitive therapy (− 1.805), and dry needling had a significant effect on pain reduction than botulinum toxin (− 1.457).

These results from the NMA should be considered with caution due to the limited quality of the evidence available and the high variability of the study protocols in terms of the method of application of dry needling and manual therapy interventions.

### Supplementary Information

**Additional file 1**. Appendix S1. Search strategy.

**Additional file 2**. Appendix S2. PEDro criteria and scores for the included trials.

**Additional file 3**. Appendix S3. Characteristics of the selected studies.

**Additional file 4**. Appendix S4.

**Additional file 5**. Appendix S5.

**Additional file 6**. Appendix S6. Baseline demographic characteristics through groups.

**Additional file 7**. Appendix S7. Contribution studies table.

## Data Availability

All data generated or analysed during this study are included in this published article (and its Additional files).
