# Supplementary material for: Effectiveness of deep dry needling versus manual therapy in the treatment of myofascial temporomandibular disorders: a systematic review and network meta-analysis
Source: Chiropr Man Therap. 2023 Nov 3;31:46. doi: 10.1186/s12998-023-00489-x (PMC10625247; doi:10.1186/s12998-023-00489-x)
Supplement: Supplementary file 1 — Additional file 1. Appendix S1. Search strategy. [file 12998_2023_489_MOESM1_ESM.docx]

**APPENDIX S1. Search strategy**

(manual ther* OR physiotherapy OR physical therapy OR dry needl* OR invasive physiotherapy OR musculoskeletal manipulation OR manipulat* OR joint mobili* OR massage OR osteopath* OR rehabilitation) AND (temporomandibular joint disorder OR craniomandibular disorder OR temporomandibular joint dysfunction OR temporomandibular joint syndrome OR myofascial pain OR trigger points OR facial pain OR TMD OR TMJ OR myofascial pain syndrome OR myofascial trigger point pain) AND “randomized control trial”.

**Search strategy PUBMED. Searched on 24/5/2020**

| **1** | manual ther* | 54.074 |
| --- | --- | --- |
| **2** | physiotherapy | 185.141 |
| **3** | physical therapy | 318.002 |
| **4** | dry needl* | 1.601 |
| **5** | invasive physiotherapy | 3.653 |
| **6** | musculoskeletal manipulation | 2.896 |
| **7** | manipulat* | 197.438 |
| **8** | joint mobili* | 13.341 |
| **9** | massage | 15.761 |
| **10** | osteopath* | 23.706 |
| **11** | rehabilitation | 629.729 |
| **12** | temporomandibular joint disorder | 18.656 |
| **13** | craniomandibular disorder | 17.571 |
| **14** | temporomandibular joint dysfunction | 6.792 |
| **15** | temporomandibular joint syndrome | 5.737 |
| **16** | myofascial pain | 3.806 |
| **17** | trigger points | 3.137 |
| **18** | facial pain | 18.508 |
| **19** | TMD | 10.730 |
| **20** | TMJ | 28.201 |
| **21** | myofascial pain syndrome | 18.349 |
| **22** | myofascial trigger point pain | 6.908 |
| **23** | randomized control trial | 303.527 |
| **24** | 1 OR 2 OR 3 OR 4 OR 5 OR 6 OR 7 OR 8 OR 9 OR 10 OR 11 | 1.045.941 |
| **25** | 12 OR 13 OR 14 OR 15 OR 16 OR 17 OR 18 OR 19 OR 20 OR 21 OR 22 | 65.830 |
| **26** | 24 AND 25 AND 23 | 726 |

**Search strategy PEDro. Searched on 23/5/2020**

| **1** | manual ther* | 1.567 |
| --- | --- | --- |
| **2** | physiotherapy | 4.394 |
| **3** | physical therapy | 6.598 |
| **4** | dry needl* | 146 |
| **5** | invasive physiotherapy | 86 |
| **6** | musculoskeletal manipulation | 4.528 |
| **7** | manipulat* | 8.323 |
| **8** | joint mobili* | 3.864 |
| **9** | massage | 8.213 |
| **10** | osteopath* | 210 |
| **11** | rehabilitation | 10.152 |
| **12** | temporomandibular joint disorder | 53 |
| **13** | craniomandibular disorder | 8 |
| **14** | temporomandibular joint dysfunction | 48 |
| **15** | temporomandibular joint syndrome | 18 |
| **16** | myofascial pain | 441 |
| **17** | trigger points | 278 |
| **18** | facial pain | 62 |
| **19** | TMD | 133 |
| **20** | TMJ | 61 |
| **21** | myofascial pain syndrome | 159 |
| **22** | myofascial trigger point pain | 127 |
| **23** | randomized control trial | 11.353 |
| **24** | (manual therapy AND 12) | 11 |
| **25** | (dry needling AND 14) | 1 |
| **26** | (2 AND 13) | 2 |

**Search strategy CINAHL. Searched on 23/5/2020**

| **1** | manual ther* | 8.494 |
| --- | --- | --- |
| **2** | physiotherapy | 43.565 |
| **3** | physical therapy | 66.675 |
| **4** | dry needl* | 664 |
| **5** | invasive physiotherapy | 20 |
| **6** | musculoskeletal manipulation | 269 |
| **7** | manipulat* | 29.931 |
| **8** | joint mobili* | 2.245 |
| **9** | massage | 22.438 |
| **10** | osteopath* | 4.814 |
| **11** | rehabilitation | 191.069 |
| **12** | temporomandibular joint disorder | 4.051 |
| **13** | craniomandibular disorder | 85 |
| **14** | temporomandibular joint dysfunction | 3.971 |
| **15** | temporomandibular joint syndrome | 4.499 |
| **16** | myofascial pain | 2.335 |
| **17** | trigger points | 2.661 |
| **18** | facial pain | 3.490 |
| **19** | TMD | 2.333 |
| **20** | TMJ | 2.717 |
| **21** | myofascial pain syndrome | 1.728 |
| **22** | myofascial trigger point pain | 1.393 |
| **23** | randomized control trial | 6.932 |
| **24** | 1 OR 2 OR 3 OR 4 OR 5 OR 6 OR 7 OR 8 OR 9 OR 10 OR 11 | 300.154 |
| **25** | 12 OR 13 OR 14 OR 15 OR 16 OR 17 OR 18 OR 19 OR 20 OR 21 OR 22 | 12.462 |
| **26** | 24 AND 25 AND 23 | 17 |

**Search strategy WOS. Searched on 23/5/2020**

| **1** | manual ther* | 115.762 |
| --- | --- | --- |
| **2** | physiotherapy | 34.891 |
| **3** | physical therapy | 553.474 |
| **4** | dry needl* | 9.104 |
| **5** | invasive physiotherapy | 687 |
| **6** | musculoskeletal manipulation | 3.177 |
| **7** | manipulat* | 602.866 |
| **8** | joint mobili* | 35.409 |
| **9** | massage | 48.031 |
| **10** | osteopath* | 11.618 |
| **11** | rehabilitation | 462.291 |
| **12** | temporomandibular joint disorder | 18.136 |
| **13** | craniomandibular disorder | 1.452 |
| **14** | temporomandibular joint dysfunction | 8.758 |
| **15** | temporomandibular joint syndrome | 6.969 |
| **16** | myofascial pain | 6.469 |
| **17** | trigger points | 39.369 |
| **18** | facial pain | 20.756 |
| **19** | TMD | 11.359 |
| **20** | TMJ | 11.334 |
| **21** | myofascial pain syndrome | 4.225 |
| **22** | myofascial trigger point pain | 2.339 |
| **23** | randomized control trial | 588.303 |
| **24** | 1 OR 2 OR 3 OR 4 OR 5 OR 6 OR 7 OR 8 OR 9 OR 10 OR 11 | 1.740.047 |
| **25** | 12 OR 13 OR 14 OR 15 OR 16 OR 17 OR 18 OR 19 OR 20 OR 21 OR 22 | 92.037 |
| **26** | 24 AND 25 AND 23 | 804 |

**Search strategy SCOPUS. Searched on 23/5/2020**

| **1** | manual ther* | 82.755 |
| --- | --- | --- |
| **2** | physiotherapy | 89.013 |
| **3** | physical therapy | 231.245 |
| **4** | dry needl* | 4.046 |
| **5** | invasive physiotherapy | 1.954 |
| **6** | musculoskeletal manipulation | 3.584 |
| **7** | manipulat* | 507.354 |
| **8** | joint mobili* | 37.947 |
| **9** | massage | 25.443 |
| **10** | osteopath* | 10.851 |
| **11** | rehabilitation | 339.880 |
| **12** | temporomandibular joint disorder | 18.179 |
| **13** | craniomandibular disorder | 1.010 |
| **14** | temporomandibular joint dysfunction | 7.427 |
| **15** | temporomandibular joint syndrome | 6.603 |
| **16** | myofascial pain | 9.703 |
| **17** | trigger points | 16.998 |
| **18** | facial pain | 21.879 |
| **19** | TMD | 13.049 |
| **20** | TMJ | 9.818 |
| **21** | myofascial pain syndrome | 7.229 |
| **22** | randomized control trial | 235.209 |
| **23** | 1 OR 2 OR 3 OR 4 OR 5 OR 6 OR 7 OR 8 OR 9 OR 10 OR 11 | 46 |
| **24** | 12 OR 13 OR 14 OR 15 OR 16 OR 17 OR 18 OR 19 OR 20 OR 21 | 979 |
| **25** | 23 AND 24 AND 22 | 3 |

**Search strategy Cochrane. Searched on 23/5/2020**

| **1** | manual ther* | 11.129 |
| --- | --- | --- |
| **2** | physiotherapy | 11.158 |
| **3** | physical therapy | 44.648 |
| **4** | dry needl* | 711 |
| **5** | invasive physiotherapy | 322 |
| **6** | musculoskeletal manipulation | 762 |
| **7** | manipulat* | 10.680 |
| **8** | joint mobili* | 2.254 |
| **9** | massage | 4.906 |
| **10** | osteopath* | 659 |
| **11** | rehabilitation | 46.381 |
| **12** | temporomandibular joint disorder | 968 |
| **13** | craniomandibular disorder | 71 |
| **14** | temporomandibular joint dysfunction | 511 |
| **15** | temporomandibular joint syndrome | 408 |
| **16** | myofascial pain | 1.936 |
| **17** | trigger points | 2.069 |
| **18** | facial pain | 2.306 |
| **19** | TMD | 690 |
| **20** | TMJ | 514 |
| **21** | myofascial pain syndrome | 1.295 |
| **22** | myofascial trigger point pain | 750 |
| **23** | randomized control trial | 574.495 |
| **24** | 1 OR 2 OR 3 OR 4 OR 5 OR 6 OR 7 OR 8 OR 9 OR 10 OR 11 | 110.171 |
| **25** | 12 OR 13 OR 14 OR 15 OR 16 OR 17 OR 18 OR 19 OR 20 OR 21 OR 22 | 6.508 |
| **26** | 24 AND 25 AND 23 | 707 |

**Search strategy Google scholar. Searched on 24/5/2020**

| **1** | manual ther* | 923.000 |
| --- | --- | --- |
| **2** | physiotherapy | 777.000 |
| **3** | physical therapy | 3.530.000 |
| **4** | dry needl* | 19.000 |
| **5** | invasive physiotherapy | 76.900 |
| **6** | musculoskeletal manipulation | 127.000 |
| **7** | manipulat* | 33.700 |
| **8** | joint mobili* | 30.700 |
| **9** | massage | 905.000 |
| **10** | osteopath* | 40.800 |
| **11** | rehabilitation | 3.540.000 |
| **12** | temporomandibular joint disorder | 88.200 |
| **13** | craniomandibular disorder | 18.100 |
| **14** | temporomandibular joint dysfunction | 69.500 |
| **15** | temporomandibular joint syndrome | 68.900 |
| **16** | myofascial pain | 75.700 |
| **17** | trigger points | 3.010.000 |
| **18** | facial pain | 2.360.000 |
| **19** | TMD | 253.000 |
| **20** | TMJ | 194.000 |
| **21** | myofascial pain syndrome | 52.200 |
| **22** | myofascial trigger point pain | 31.600 |
| **23** | randomized control trial | 2.510.000 |
| **24** | 1 OR 2 OR 3 OR 4 OR 5 OR 6 OR 7 OR 8 OR 9 OR 10 OR 11 | 12.200 |
| **25** | 12 OR 13 OR 14 OR 15 OR 16 OR 17 OR 18 OR 19 OR 20 OR 21 OR 22 | 3.630 |
| **26** | 24 AND 25 AND 23 | 837 |

**Search strategy EMBASE. Searched on 2/6/2020**

| **1** | manual ther* | 77.845 |
| --- | --- | --- |
| **2** | physiotherapy | 137.906 |
| **3** | physical therapy | 433.206 |
| **4** | dry needl* | 2.403 |
| **5** | invasive physiotherapy | 3.185 |
| **6** | musculoskeletal manipulation | 1.895 |
| **7** | manipulat* | 235.576 |
| **8** | joint mobili* | 34.848 |
| **9** | massage | 23.808 |
| **10** | osteopath* | 34.195 |
| **11** | rehabilitation | 603.999 |
| **12** | temporomandibular joint disorder | 15.679 |
| **13** | craniomandibular disorder | 1.603 |
| **14** | temporomandibular joint dysfunction | 3.634 |
| **15** | temporomandibular joint syndrome | 2.179 |
| **16** | myofascial pain | 9.698 |
| **17** | trigger points | 4.711 |
| **18** | facial pain | 20.793 |
| **19** | TMD | 16.681 |
| **20** | TMJ | 13.458 |
| **21** | myofascial pain syndrome | 3.241 |
| **22** | myofascial trigger point pain | 1.600 |
| **23** | randomized control trial | 241.224 |
| **24** | 1 OR 2 OR 3 OR 4 OR 5 OR 6 OR 7 OR 8 OR 9 OR 10 OR 11 | 687.853 |
| **25** | 12 OR 13 OR 14 OR 15 OR 16 OR 17 OR 18 OR 19 OR 20 OR 21 OR 22 | 1.711 |
| **26** | 24 AND 25 AND 23 | 72 |
