# Supplementary material for: Effectiveness of deep dry needling versus manual therapy in the treatment of myofascial temporomandibular disorders: a systematic review and network meta-analysis
Source: Chiropr Man Therap. 2023 Nov 3;31:46. doi: 10.1186/s12998-023-00489-x (PMC10625247; doi:10.1186/s12998-023-00489-x)
Supplement: Supplementary file 2 — Additional file 2. Appendix S2. PEDro criteria and scores for the included trials. [file 12998_2023_489_MOESM2_ESM.docx]

**APPENDIX S2. *PEDro criteria and scores for the included trials.***

| STUDY | ITEMS  1 / 2 / 3 / 4 / 5 / 6 / 7 / 8 / 9 / 10 / 11 | TOTAL |
| --- | --- | --- |
| López-Martos et al., 2018(30) | 1 / 1 / 0 / 1 / 0 / 0 / 0 / 1 / 1 / 1 / 1 | 6 |
| González-Pérez et al., 2015(29) | 1 / 1 / 0 / 1 / 0 / 0 / 0 / 1 / 0 / 1 / 1 | 5 |
| Kütük et al., 2019(34) | 1 / 1 / 1 / 1 / 0 / 0 / 0 / 1 / 1 / 1 / 0 | 6 |
| McMillan et al., 1997(37) | 1 / 1 / 0 / 1 / 0 / 0 / 1 / 0 / 0 / 1 / 1 | 5 |
| Dıraçoğlu et al., 2012(38) | 1 / 1 / 0 / 1 / 1 / 1 / 1 / 1 / 1 / 1 / 1 | 8 |
| Fernández-Carnero et al., 2010(39) | 1 / 1 / 1 / 1 / 1 / 1 / 1 / 0 / 1 / 1 / 1 | 9 |
| Silva et al., 2012(40) | 1 / 1 / 0 / 1 / 1 / 0 / 1 / 1 / 0 / 1 / 1 | 8 |
| Oliveira Campelo et al., 2010(41) | 1 / 1 / 0 / 1 / 0 / 0 / 1 / 1 / 0 / 1 / 1 | 6 |
| Ibañez-Garcia et al., 2009(42) | 0 / 1 / 1 / 1 / 0 / 0 / 1 / 0 / 1 / 1 / 1 | 7 |
| De Laat et al., 2003(35) | 1 / 1 / 0 / 1 / 0 / 0 / 1 / 0 / 0 / 1 / 1 | 5 |
| Kalamir et al., 2013(36) | 1 / 1 / 1 / 1 / 0 / 0 / 1 / 1 / 0 / 1 / 1 | 7 |
| Shousha et al., 2008(43) | 1 / 1 / 1 / 1 / 0 / 0 / 1 / 0 / 0 / 1 / 1 | 6 |
| Corum et al., 2018(32) | 1 / 1 / 1 / 1 / 0 / 0 / 1 / 1 / 1 / 1 / 1 | 8 |
| Guarda-Nardini et al., 2012(33) | 1 / 1 / 0 / 1 / 0 / 0 / 0 / 0 / 0 / 1 / 1 | 4 |
| Reynolds et al., 2020(31) | 1 / 1 / 1 / 1 / 0 / 0 / 1 / 1 / 1 / 1 / 1 | 8 |
| La Touche et al., 2013(44) | 1 / 1 / 1 / 1 / 1 / 0 / 1 / 1 / 0 / 1 / 1 | 8 |
| Rodriguez-Blanco et al., 2015(45) | 0 / 1 / 0 / 1 / 1 / 0 / 1 / 1 / 1 / 1 / 1 | 8 |

1, selection criteria; 2, random allocation; 3, concealed allocation; 4, groups similar at baseline; 5, participant blinding; 6, therapist blinding; 7, assessor blinding; 8, <15% dropouts; 9, intention to-treat-analysis; 10, between-group difference reported; 11, point estimate and variability reported.

Item 1 does not contribute to the total score.
