# Supplementary material for: Effectiveness of deep dry needling versus manual therapy in the treatment of myofascial temporomandibular disorders: a systematic review and network meta-analysis
Source: Chiropr Man Therap. 2023 Nov 3;31:46. doi: 10.1186/s12998-023-00489-x (PMC10625247; doi:10.1186/s12998-023-00489-x)
Supplement: Supplementary file 3 — Additional file 3. Appendix S3. Characteristics of the selected studies. [file 12998_2023_489_MOESM3_ESM.docx]

| AUTHORS | SAMPLE | GROUPS | THERAPY | Nº SESSIONS | FOLLOW-UP | VARIABLES | RESULTS |
| --- | --- | --- | --- | --- | --- | --- | --- |
| López-Martos et al., 2018 (30) | N=20  N=20  N=20 | PNE  DDN  SNP (control) | DDN lateral pterygoid | 3 (1/week)  3 (1/week)  3 (1/week) | Day 0  Day 28  Day 42  Day 70 | Age  Gender  Pain at rest  Pain mastication  MIO  TMJ functionality test | Significant differences (*p* <0.01) for the PNE and DDN groups with respect to pain reduction at rest, during chewing, and for MIO. Significantly earlier improvement for the PNE group. Differences for PNE and DDN groups with respect to SNP group were significant (*p* <0.05) up to day 70. |
| González-Pérez et al., 2015 (29) | N=24  N=24 | DDN  Paracetamol (control) | DDN lateral pterygoid | 3 (1/week)  2 tablets every 6h/3 week | Day 0  Day 28  Day 70 | Age  Gender  Pain at rest  Pain mastication  Jaw opening  Left lateral mov  Right lateral mov  Protrusion mov | Significant difference (*p*<0.05) for both groups with respect to pain reduction at rest and with mastication, but the DDN group had better levels of pain reduction. Significant differences (*p*<0.05) up to day 70 in the DDN respect to jaw opening, laterality and protrusion movements compared with pretreatment. |
| Kütük et al., 2019 (34) | N=20  N=20 | DDN  Botulinum toxin-A | DDN masticatory muscles | 1  1 | Day 0  Week 6 | Age  Gender  Side of involvement  Pain mastication  Pain at rest  Crepitation  Jaw opening  Functional limitation  Jaw strength  Muscle spasm  Protrusion mov  Left lateral mov  Right lateral mov | Difference between groups regarding VAS for TMJ pain at rest (P=0.048). The pain at rest was more effectively in Group 2 at the end of 6 weeks. Improvement in right (P=0.009) and left (P=0.002) protrusion was more evident in Group 2 after 6 weeks. In Group 2, the TMJ function was more obvious in 6 weeks following dry needling (P=0.002). |
| McMillan et al., 1997 (37) | N=10  N=10  N=10 | Procaine + simulated DN  DN + simulated LA  Simulated LA + simulated DN (control) | DN masseter | 3 (1/week)  3 (1/week)  3 (1/week) | Week 1 (Pre, 5 mins, 1h and 24h)  Week 2 (Pre, 5 mins, 1h and 24h)  Week 3 (Pre, 5 mins, 1h and 24h) | PPT masseter  PPT temporalis  Pain intensity  Pain unpleasantness  Visual stimulus | PPT increased slightly after treatment in all groups. Pain intensity and unpleasantness decreased significantly at the end in all groups. No significant between-group differences in PPT and VAS at the end of the treatment. |
| Dıraçoğlu et al., 2012 (38) | N=26  N=26 | DN  SDN (control) | DN masseter and temporalis | 3 (1/week)  3 (1/week) | Pre  1 week post | Age  Gender  Occupation  PPT  VAS  Unassisted jaw opening without pain | Values were higher in the study group (p<0,05). No differences in terms of VAS and unassisted jaw-opening without pain values. |
| Fernández-Carnero et al., 2010 (39) | N=12 | DN  SDN (control) | DN masseter | 2 (1/week) | Pre  5 mins post | Age  PPT masseter  PPT condyle  Jaw opening | Interaction between intervention and time for PPT levels in the masseter (p<0,001) and condyle (p<0,001), and pain-free active mouth opening (p<0,001). Improvements in all the DDN outcomes (p<0,001). |
| Silva et al., 2012 (40) | N=8  N=8 | DN  Lidocaine | DN masticatory muscles | 1  1 | Pre  10 mins  24h  Day 7 post  Day 15 post  Day 21 post  Day 30 post | PPT  VAS | No differences for PPT, but for all groups the PPT during the time increased when compared the pretreatment. VAS showed differences between groups and during the time. Lidocaine has the lowest VAS values, but at 30 days there were no differences. |

| AUTHORS | SUBJECTS | GROUPS | THERAPY | Nº SESSIONS | FOLLOW-UP | VARIABLES | RESULTS |
| --- | --- | --- | --- | --- | --- | --- | --- |
| Oliveira Campelo et al., 2010(41) | N=41  N=41  N=40 | Manipulative  Soft tissue  Control (no intervention) | Atlanto-occipital joint thrust  Suboccipital muscle inhibition | 1  1 | Pretto  2 mins post | Age  Body mass  Height  PPT masseter  PPT temporalis  Active mouth opening | Significant group-by-time interaction for changes in PPT over masseter (P<.01) and temporalis (P=.003) muscle latent TrPs and for active mouth opening (P<.001) in the manipulative and soft tissue groups. Between-group effect sizes were small. |
| Ibañez-Garcia et al., 2009(42) | N=22  N=25  N=24 | MT  MT  Control (no intervention) | Neuro-muscular technique  Strain-counterstrain | 3 (1/week)  3 (1/week) | Pre  1 week | Age  Geder  PPT  Active mouth opening  Local pain | Significant group x time interaction for changes in PPT, changes in active mouth opening, and local pain. Effect sizes were large for PPT and mouth opening, and moderate for local pain in both intervention groups; but small for the control. No significant differences between both intervention groups. |
| De Laat et al., 2003(35) | N=13  N=13 | MT  MT | Heat application, massage, ultra-sound and muscle stretching (both groups) | 3/week in 4 week  3/week in 6 week | Pretto  2 week  4 week  6 week | Present pain  Lowest and highest pain over the past period  % of pain relief  Jaw function assessment by the MFIQ  PPT masseter  PPT temporalis  PPT thumb muscles | Pain and MFIQ decreased while PPTs increased in both groups. After 4 and 6 weeks were significant differences for the PPT of the masseter in group I (P<.02) and the temporalis in both groups (P<.01). VAS of present (P<.02), min (P<.01), and max (P<.0001) pain and the MFIQ (P<.001) improved. After 6 weeks, a mean of 60% pain decrease was reported (P<.0001). No differences between the groups receiving 4 weeks vs 6 weeks of physical therapy. |
| Kalamir et al., 2013(36) | N=23  N=23 | MT  Exercise and education | Intraoral myofascial | 2/week in 5 week | Pre  6 week | Age  Gender  Pain at rest  Pain on opening  Pain on clenching  Opening range | IMT had lower average pain for all primary outcomes at  6 weeks compared to the ESC group (p<0.001). The IMT group had higher odds of a clinically significant change (p<0.045). No difference in opening range between the groups. Both groups achieved significant decreases in all three pain measures at six weeks (p≤0.05), but only the IMT achieved changes of 2 or more points. |
| Shousha et al., 2008(43) | N=56  N=56 | MT  Occlusive splinting | Relaxed jaw position and stretching | 2/week in 6 week  6 week | Pre  6 week | Age  Gender  Weight  BMI  Pain  TMJ opening index | Significant reduction in pain intensity and TMJ opening index in favor of the conservative physiotherapy group. |
| Corum et al., 2018(32) | N=20  N=20  N=20 | MT  Sham MT + exercise  Education (control) | Manipulation + exers | 6  6 | Pre  Post  1 month | Age  BMI  Pain duration  Neck pain  Headache  Pain  PPT masetter  PPT temporalis  Pain-free MMO  Quality of life | Significant differences in the CSM+NE group vs. the SM+NE and PE groups posttreatment. Although PPT increased significantly in the CSM+NE group, no significant changes in  any PPT were found in either the SM+NE or PE group. Regarding pain-free MMO and SF-36 scores, there were significant increases posttreatment in the CSM+NE and SM+NE groups compared to the PE group. |
| Guarda-Nardini et al., 2012(33) | N=15  N=15 | MT  Botulinum toxin | Fascial manipulation | 3/week in 2-4 week  1 | Pre  Post  3 months | Age  Gender  Pain  Mouth opening  Left laterotrusion  Protrusion  Right laterotrusion | Both protocols provided significant improvement over time for pain symptoms. The two treatments seem to be almost equally effective, Fascial Manipulation being slightly superior to reduce subjective pain perception, and botulinum toxin injections being slightly superior to increase jaw range of motion. |
| Reynolds et al., 2020(31) | N=25  N=25 | MT  Sham MT | Thrust, education, soft tissue mobilization (suboccipital muscle inhibition) and exercise | 1/week in 4 week  (both groups) | Pre  Post 1ºss  1 week  4 week | Age  Gender  BMI  Duration pain  Jaw pain  Neck pain  Headache  Jaw range of motion  Cervical ROM  Maximal mouth opening  Kinesiophobia  Jaw functional limitation  Neck disability  Temporomandibular disability | There was no significant interaction for MMO, NPRS, or secondary measures. Significant 2-way interactions were noted in JFLS and TSK-TMD. The HVLAT group had lower fear at 4-weeks and improved jaw function earlier. GROC favored the HVLAT group with significant differences in successful outcomes noted immediately after baseline treatment and at 4-weeks. |
| La Touche et al., 2013(44) | N=16  N=16 | MT  Sham MT | Mobilization of the upper cervical spine | 3ss in 2 week | Pre  Post 2ºss  Post tto | Age  Anxiety  Depression  Neck disability  Pain intensity  PPT  HRV | The PPT in the craniofacial and cervical regions significantly increased (P < 0.001) and pain intensity significantly decreased (P<0.001) in the treatment group compared with placebo. |
| Rodriguez-Blanco et al., 2015(45) | N=30  N=30 | MT  Control | Neuromuscular technique masseter and passive hamstring stretching + suboccpital inhibition  Neuromuscular technique masseter and passive hamstring stretching | 1  1 | Pret  Post | Age  Gender  Height  Weight  PPT masseter  PPT trigeminal nerve  ROM suboccipital  Lumbar mobility  Vertical mouth opening | EG observed an increase in suboccipital flexion and the SAR test. No significant differences were found in the between-group comparison for any variable (p > 0.05). |

**APPENDIX S3. Characteristics of the selected studies.**
