# Supplementary material for: Effectiveness of deep dry needling versus manual therapy in the treatment of myofascial temporomandibular disorders: a systematic review and network meta-analysis
Source: Chiropr Man Therap. 2023 Nov 3;31:46. doi: 10.1186/s12998-023-00489-x (PMC10625247; doi:10.1186/s12998-023-00489-x)
Supplement: Supplementary file 4 — Additional file 4. Appendix S4. [file 12998_2023_489_MOESM4_ESM.docx]

**Appendix S4.**

|  | **Difference direct-indirect estimates** | **Z-score** |
| --- | --- | --- |
| Botulinum toxin vs Deep dry needling | -1.331±1.403, 95%CI (-4.08, 1.419) | Z=-0.948, p=0.343 |
| Botulinum toxin vs Manual therapy | 1.331±1.403, 95%CI (-1.419, 4.08) | Z=0.948, p=0.343 |
| Cognitive therapy vs Manual therapy | 0.703±2.301, 95%CI (-3.808, 5.214) | Z=0.305, p=0.76 |
| Cognitive therapy vs Placebo | 0.639±1.158, 95%CI (-1.632, 2.909) | Z=0.551, p=0.581 |
| Deep dry needling vs Placebo | -1.331±1.403, 95%CI (-4.08, 1.419) | Z=-0.948, p=0.343 |
| Manual therapy vs Placebo | 0.208±1.223, 95%CI (-2.189, 2.604) | Z=0.17, p=0.865 |
