# Supplementary material for: Effectiveness of deep dry needling versus manual therapy in the treatment of myofascial temporomandibular disorders: a systematic review and network meta-analysis
Source: Chiropr Man Therap. 2023 Nov 3;31:46. doi: 10.1186/s12998-023-00489-x (PMC10625247; doi:10.1186/s12998-023-00489-x)
Supplement: Supplementary file 5 — Additional file 5. Appendix S5. [file 12998_2023_489_MOESM5_ESM.pptx]

## Slide 1
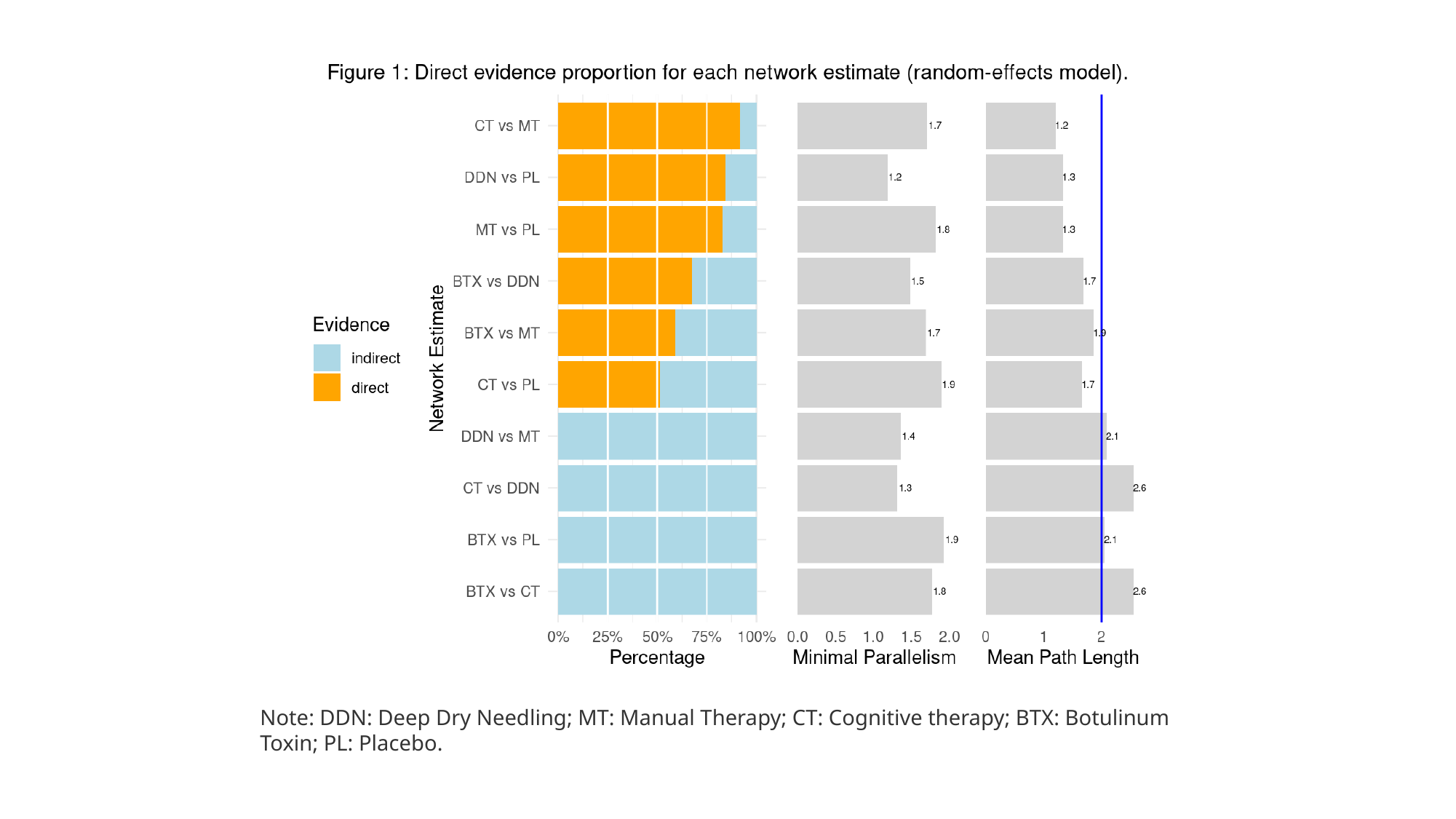

Note: DDN: Deep Dry Needling; MT: Manual Therapy; CT: Cognitive therapy; BTX: Botulinum Toxin; PL: Placebo.
