# Supplementary figures and images for: Effectiveness of deep dry needling versus manual therapy in the treatment of myofascial temporomandibular disorders: a systematic review and network meta-analysis
Source: Chiropr Man Therap. 2023 Nov 3;31:46. doi: 10.1186/s12998-023-00489-x (PMC10625247; doi:10.1186/s12998-023-00489-x)

| **APPENDIX S6. Baseline demographic characteristics through groups.** |
| --- |


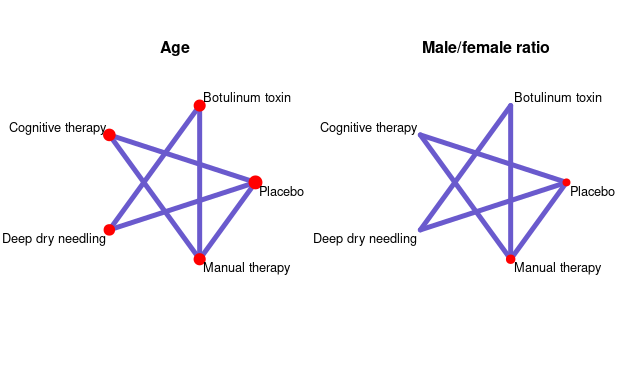

Supplement: Supplementary file 6 — Additional file 6. Appendix S6. Baseline demographic characteristics through groups. [file 12998_2023_489_MOESM6_ESM.docx]

| **Appendix S7. Contribution studies table.** |
| --- |


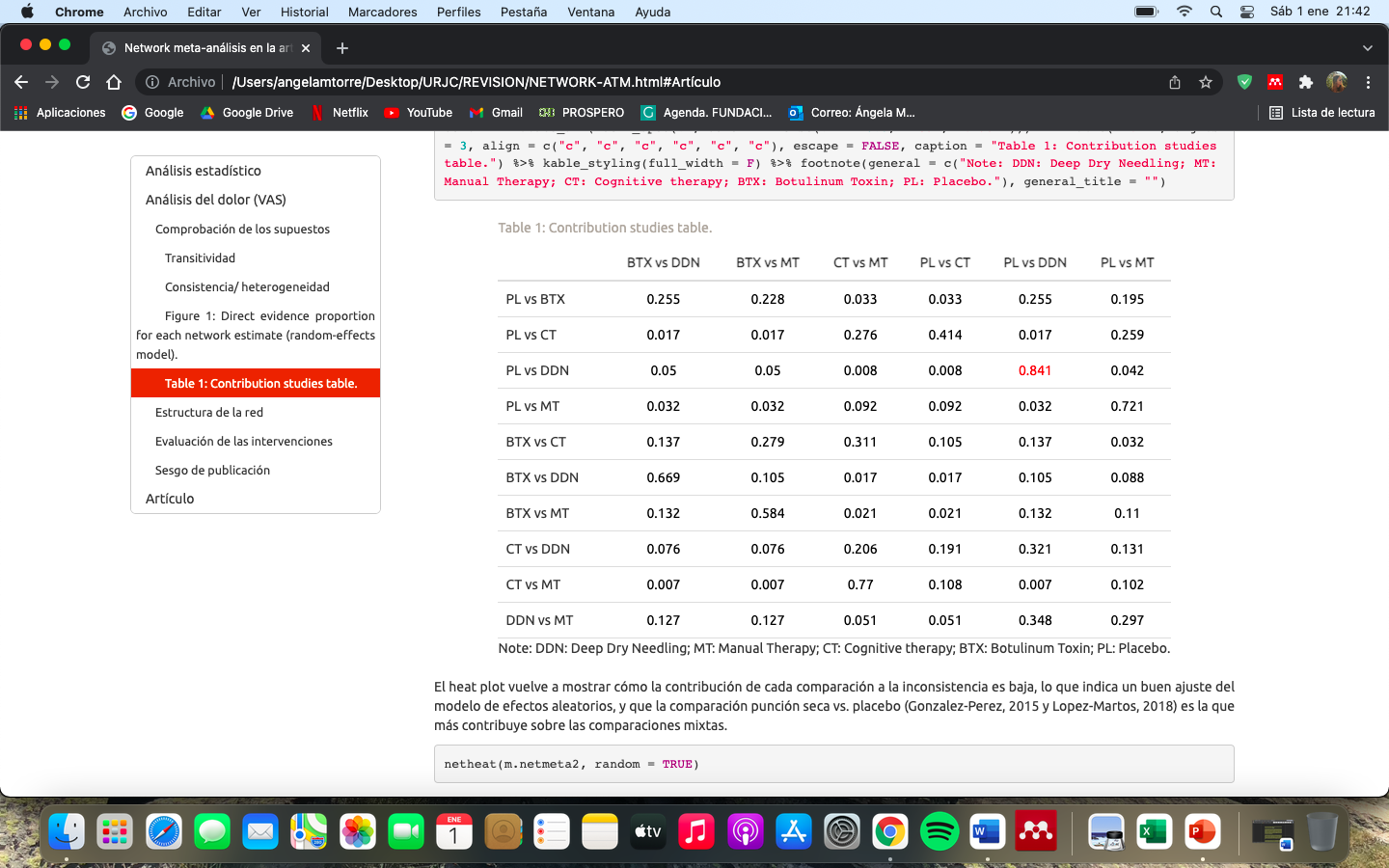

Supplement: Supplementary file 7 — Additional file 7. Appendix S7. Contribution studies table. [file 12998_2023_489_MOESM7_ESM.docx]
